# Four-Dimensional Printing of Multi-Material Origami and Kirigami-Inspired Hydrogel Self-Folding Structures

**DOI:** 10.3390/ma17205028

**Published:** 2024-10-15

**Authors:** Divambal Appavoo, Nilab Azim, Maged Elshatoury, Dennis-Xavier Antony, Swaminathan Rajaraman, Lei Zhai

**Affiliations:** 1 NanoScience Technology Center, University of Central Florida, Orlando, FL 32826, USA; 2 Department of Chemistry, University of Central Florida, Orlando, FL 32826, USA; 3 Department of Materials Science and Engineering, University of Central Florida, Orlando, FL 32816, USA; 4 Department of Electrical and Computer Engineering, University of Central Florida, Orlando, FL 32816, USA; 5 Burnett School of Biomedical Sciences, University of Central Florida, Orlando, FL 32816, USA

**Keywords:** hydrogel, poly(N-iso-propylacrylamide), 4D printing, stimuli responsive, double layer

## Abstract

Four-dimensional printing refers to a process through which a 3D printed object transforms from one structure into another through the influence of an external energy input. Self-folding structures have been extensively studied to advance 3D printing technology into 4D using stimuli-responsive polymers. Designing and applying self-folding structures requires an understanding of the material properties so that the structural designs can be tailored to the targeted applications. Poly(N-iso-propylacrylamide) (PNIPAM) was used as the thermo-responsive material in this study to 3D print hydrogel samples that can bend or fold with temperature changes. A double-layer printed structure, with PNIPAM as the self-folding layer and polyethylene glycol (PEG) as the supporting layer, provided the mechanical robustness and overall flexibility to accommodate geometric changes. The mechanical properties of the multi-material 3D printing were tested to confirm the contribution of the PEG support to the double-layer system. The desired folding of the structures, as a response to temperature changes, was obtained by adding kirigami-inspired cuts to the design. An excellent shape-shifting capability was obtained by tuning the design. The experimental observations were supported by COMSOL Multiphysics^®^ software simulations, predicting the control over the folding of the double-layer systems.

## 1. Introduction

Four-dimensional (4D) printing technologies involve the three-dimensional (3D) printing of responsive materials that are capable of undergoing a configuration (e.g., size) and/or property change (e.g., modulus) over time, triggered by external stimuli from either environmental or human intervention, including temperature and electricity [1,2,3,4]. Four-dimensional printing advances 3D printing for soft robotics development where environmental stimulation, including temperature, pH, light and magnetic field, changes the shape of the robotic parts [5,6]. Therefore, the combination of advanced 3D printing technology and the choice of material is crucial to obtain a structure with the desired properties for soft robotics applications [6,7]. For example, digital light printing (DLP) is a 3D printing technique based on Vat Polymerization that produces rapid and high-resolution printing, which is an important aspect, especially for microstructures with intricate designs [8,9]. Multi-material 3D printing is a recent approach that involves printing materials with different functionalities to create complex, heterogeneous printed objects that have innovative applications [10,11,12,13,14]. However, the multi-material 3D printed structures obtained through DLP are limited to photocurable acrylamide-polyethylene diacrylate (PEGDA)-based [15,16] and polymethyl methacrylate (PMMA)-based resins [17]. Four-dimensional printing has been developed by several researchers using responsive materials [18], and various experimental and simulated mechanisms of folding have been reported in the literature [19].

Stimuli-responsive polymers have become an important class of polymers in the development of smart materials by undergoing a chemical/physical change in their properties upon exposure to an external stimulus [20,21,22,23]. Poly(N-iso-propylacrylamide) (PNIPAM) is a thermo-responsive polymer that has been extensively explored for biomedical applications [20,22,24,25]. It is biocompatible and undergoes a large and reversible volume change at its lower critical solution temperature (LCST), around 32 °C [26,27]. In the reported attempts at 4D printing using PNIPAM [28,29,30,31,32], the poor mechanical properties of PNIPAM have hindered its potential applications and have required chemical modifications, such as crosslinking with a higher-density polymer, to increase its mechanical robustness [33]. Wang et al. recently produced a bilayer system via a direct-ink printing technique and reported the good responsiveness and mechanical properties of the structures [34]. The bilayer system they reported on consisted of a PNIPAM:PEG layer thickness ratio of 2:1, which makes the structure more prone to bending upon temperature changes, compromising its mechanical properties.

The centuries-old origami and kirigami paper-folding art forms have been adopted in the designing of self-folding structures that allow for responsive polymer samples to change their shapes with external stimuli. For example, kirigami cuts increase the structure flexibility of built constructs upon stretching. The positioning of the cuts in a 2D structure governs the final geometries and, hence, can be tailored for targeted deformations [35,36,37]. The fabrication of origami and kirigami structures using 3D printing has generated more elaborate and complex material designs and broadened the application of self-folding structures [38], including grippers [39,40] and self-folding boxes [41], among others. It is expected that the combination of advanced 3D printing technology, kirigami-inspired design and stimuli-responsive multi-materials will introduce flexibility and versatility to printed objects for various novel applications.

This research focuses on establishing a new strategy involving the fusion of different design concepts into a single model to maximize shape transformation, such as a combination of double-layered structures with kirigami cuts. We 3D printed a smart multi-material object consisting of a double-layer system, composed of a thermo-responsive PNIPAM layer and a non-responsive PEG layer with kirigami cuts. In this study, the bilayer models for the different kirigami designs were printed with equal thicknesses of PNIPAM and PEG using a DLP 3D printer. The folding behavior, thermal responsiveness and mechanical robustness of the structures were studied to understand the degree of structural flexibility for potential applications, such as soft robotics building blocks.

## 2. Materials and Methods

PNIPAM and N-isopropylacrylamide (NIPAM) were obtained from Polysciences Inc. (Warrington, PA, USA), and poly(ethylene glycol) dimethacrylate (PEGDMA) and diphenyl (2,4,6-trimethylbenzoyl) phosphine oxide (TPO) were obtained from Sigma Aldrich (St. Louis, MO, USA). n-Butanol and isopropyl alcohol (IPA) were obtained from Fischer Scientific (Hampton, NH, USA).

Preparation of PNIPAM pre-polymer resin: The PNIPAM pre-polymer solution was prepares as follows. To an n-butanol (50 mL) solution of PNIPAM (2.80 g, Mn ~ 40 kDa), NIPAM (21.40 g, 0.18 moles) was added and stirred until the solution turned clear. Crosslinker PEGDMA (1.43 g, Mn ~ 550 Da) and photoinitiator TPO (0.10 g, 0.27 mmol) were added to the above solution and stirred at room temperature in the dark to obtain the pre-polymer resin of PNIPAM.

Preparation of PEG pre-polymer resin: a 2.5 wt% TPO/PEG was prepared by stirring TPO (1.28 g, 3.65 mmol) in PEGDMA (50.00 g, Mn ~ 550 Da) for ~2 h in the dark.

Three-dimensional printing: The self-folding sheets were printed using an Asiga MAX X27 printer (Asiga Ltd., Alexandria, NSW, Australia) that uses the DLP technique using a UV light of 385 nm and a power density of 29 mW/cm^2^. The building volume for the Asiga MAX X27 printer is 29.2 mm × 51.8 mm × 75 mm and the printer has a pixel resolution of 27 μm in X and Y with a Z resolution of ~1 μm. A high-power UV LED provided the photocuring light of 385 nm wavelength.

The printing parameters were optimized for each pre-polymer system and are summarized in Table S1. PEG was printed as the first layer. The printed structure was then rinsed with IPA and dried in air before printing the second layer. The printing parameters were changed, and the pre-polymer bath was switched for printing the PNIPAM layer. After both layers had been printed, the structure was removed from the printing stage and ultra-sonicated in IPA five minutes.

Thermal responsiveness: The printed structures were allowed to equilibrate in distilled water at room temperature for 24 h. To test the folding/unfolding of the printed structures, the swollen structures were transferred from hot water (~50 °C) to cold water (~15 °C) and vice versa, leaving the structures to equilibrate at that temperature for ~15 min before recording the measurements.

Microstructure analysis: The microstructure of the 94 wt% PNIPAM hydrogel was analyzed using optical microscopy and a scanning electron microscope (SEM). The sample was prepared by first immersing the crosslinked polymer mixture (94 wt% PNIPAM) in water for 24 h at room temperature to ensure the complete hydration of the polymer chains. The swollen hydrogel was subsequently stored overnight in the freezer to solidify. The frozen hydrogel was then freeze-dried for two days in a lyophilizer. The surface and the cross section of the sample were examined by an SEM using a Zeiss ULTRA-55 FEG SEM (Carl Zeiss AG Oberkochen, Baden-Württemberg, Germany). The sample was gold-coated using a sputter coater before SEM imaging.

Mechanical testing: A dynamic mechanical analyzer, Discovery DMA850, was used for testing the dynamic mechanical properties of the PNIPAM and PEG single- and double-layer structures. Static compression tests were carried out at room temperature (~20 °C), and dynamic compression tests were performed within a temperature range of ~23–40/60 °C, with heating ramp rates of 1 and 3 °C/min. The swollen disks were placed between the compression clamps and a preload force of 2.0 N was used. The dimensions for the single- (PNIPAM) and double-layer (PNIPAM/PEG) structures are provided in the supporting information (Figure S8A). For the static tensile strength measurement, the testing sample was designed such that the PNIPAM was in the middle and connected to two PEG support structures on each side, that would later be mounted onto the tensile clamps (Figure S8B). Due to the limitations of the DMA850, which allows for the testing of mostly hydrogels, the tensile strength of the crosslinked PEGDMA was measured using an Instron, and the sample was prepared following the ASTM D638 [42] Type D dog-bone shape (see Figure S5). Each experiment was repeated at least three times and the average is reported with the standard deviation.

Computational simulation: COMSOL Multiphysics^®^ software V5.4 licensed by the University of Central Florida was used to perform the modeling experiment in a 3D plane. Previously shown to efficiently report the simulation of hydrogels [43], the Heat Transfer in Solids module was used to simulate the deformation of the bilayer system based on a time-dependent study. A linear elastic material model was used for the solid mechanics study, with the isotropic nature of the material assumed. A fine mesh size was generated for better results. The dimensions for the models used are given in Table S2, and the parameters used for the simulations are provided in Table S3. The coefficient of the thermal expansion of PNIPAM is shown in Figure S10.

## 3. Results and Discussion

Although PNIPAM-based thermally triggered self-folding structures have been extensively investigated [20,22,44], their applications have been limited by their low mechanical strength [33,45]. Among the strategies reported for improving the mechanical strength of PNIPAM, the most popular one involves the crosslinking of PNIPAM with other polymers [33]. Such copolymers are stronger than homopolymer PNIPAM; however, their degree of thermal response is compromised due to the lower PNIPAM content in the polymer mixture [46].

We took a different approach to obtain a mechanically robust structure based on a PNIPAM hydrogel while maintaining the excellent thermal response of PNIPAM. In addition to a mechanically strong 3D printed structure, the objective of this study was to 3D print a self-folding object. Typically, self-folding structures/sheets consist of two materials with different properties, and an inhomogeneous volume change causes the bending of the structure [47]. In this research work, the self-folding effect of a double-layer polymer-based structure with one active and one passive layer was studied. PNIPAM was chosen as the active layer and biocompatible PEG as the passive layer, obtained by crosslinking poly(ethylene glycol) dimethacrylate (PEGDMA) in the presence of the photoinitiator diphenyl(2,4,6-trimethylbenzoyl) phosphine oxide (TPO). This double-layer structure was expected to result in an overall stronger structure, with the PEG acting as a stronger backbone to the PNIPAM layer, while at the same time displaying some degree of flexibility to accommodate the volume changes in the PNIPAM. The PEG layer, hence, helps incorporate mechanical strength into the double-layer structure while avoiding cracks upon folding [48,49].

**Figure 1:**
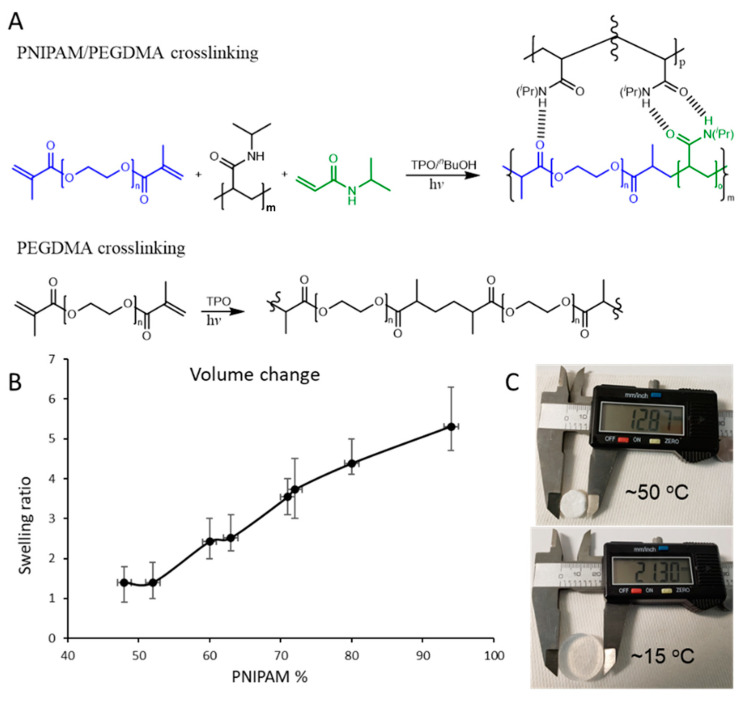
Reaction schemes for PNIPAM/PEG and PEGMA crosslinking (**A**). Plot of swelling ratio versus wt% PNIPAM in polymer mixture (**B**). Photographs of 94 wt% PNIPAM disk in swollen and collapsed states (**C**).

The PNIPAM layer was slightly modified to improve its mechanical strength by adding PEGDMA to the pre-polymer matrix. The chemical crosslinking took place in the presence of the TPO photoinitiator when exposed to 365 nm UV light at an intensity of 4.5 mW/cm^2^. Figure 1A shows the reaction schemes of the crosslinking reactions in the PEGDMA and the PNIPAM resins. In the PNIPAM layer, the NIPAM polymerizes and couples with the crosslinking agent, PEGDMA. The PNIPAM/PEG chains interact with the PNIPAM polymer chains via intermolecular H-bonding. The exact chemical composition of the polymer matrix was not investigated in this study. Samples were prepared with different PNIPAM:PEG content ratios, and their volumetric changes as a function of temperature (T < LCST and T > LCST) were recorded (Figure 1B). A maximum response of a 1:6.3 swelling ratio was obtained for a 94 wt% of PNIPAM in the polymer mixture (Figure 1C). While the PEG’s concentration in the matrix was enough to strengthen the PNIPAM, its influence on the thermal responsiveness of the PNIPAM was negligible. The swelling ratio of the crosslinked PEGDMA was found to be insignificant and, hence, was not considered in this study.

Ge et al. recently reported the multi-material 3D printing of an acrylate-based highly stretchable 3D hydrogel structure using the DLP printing technique [15]. Their method involved the printing of one layer and then directly switching to the next resin. The strong attachment of the second resin to the printed layer likely resulted from a crosslinking reaction between the residual resin from the first print and the second resin. However, the composition of the interface cannot be controlled and may result in the inconsistent adhesion of the two layers, making it difficult to reproduce. To make the multi-material 3D printing process using a DLP printer more consistent, we performed double-layer printing in a slightly different manner. We first optimized the printing parameters for each resin separately to obtain the best resolution and adhesion of one polymer to the other. For instance, while the burn-in and exposure times are 1.5 s each for PEG, the PNIPAM resin does not print under 10 s of exposure and burn-in times. The separation velocity and separation distance also played a role in the printing process. However, unlike the burn-in and exposure times, the separation velocity and distance were the same for both resins.

**Figure 2:**
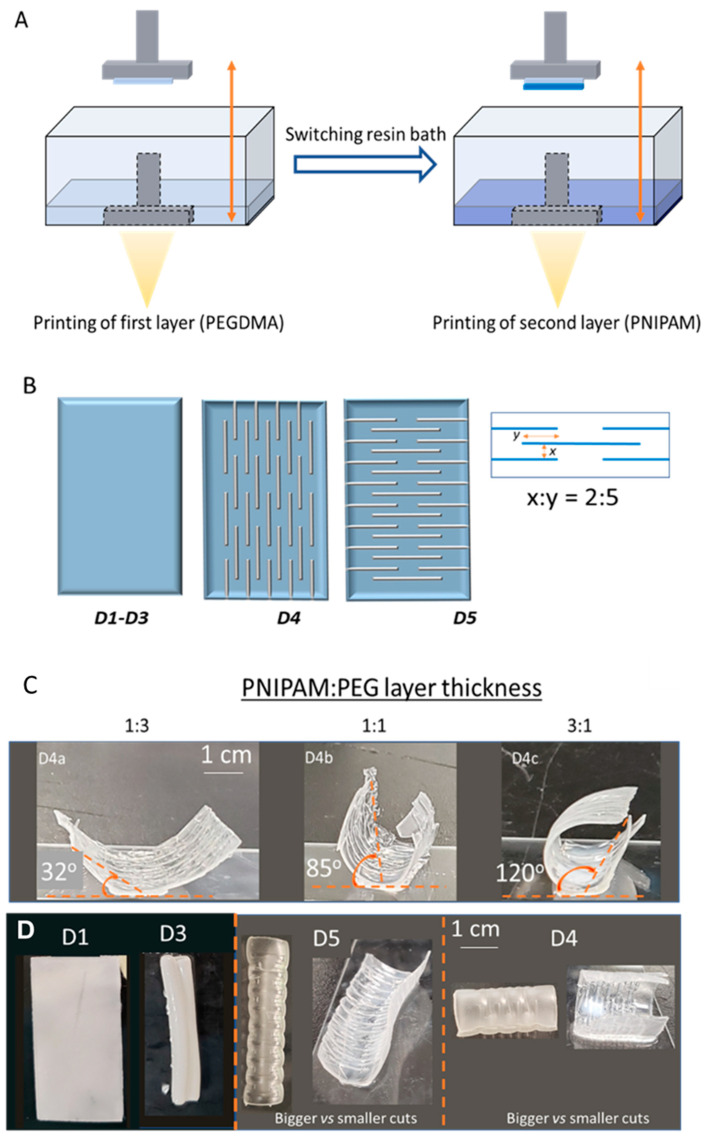
DLP printing of double polymer system (**A**). Design of double-layer sheet (**B**). Degree of folding of D4 (at T ~ 50 °C) with varying PNIPAM:PEG thickness in double-layer sheets (**C**). Temperature responsiveness of D1, D3, D4 and D5 sheets (with big and smaller cuts) at ~50 °C (**D**).

The order in which the polymers were printed was then assessed to obtain the optimum stability of the double-layer structure. We found that the first printed layer determined how well the second layer was attached to the printed object and, for the two polymer systems used in this research, PEG must be the first printed layer for a stable structure (Figure 2A). When PNIPAM was printed first, the PEG layer had difficulty attaching to the PNIPAM printed structure and, despite several attempts to change the printing parameters, incomplete structures were obtained. This might have been because the PNIPAM was too soft to support the PEG layer, causing either the partial attachment of the PEG to the PNIPAM layer or the complete delamination of the PEG layer into the resin bath. On the other hand, when printed as the first layer, the PEG provided a sturdy backbone that could support the weight of the PNIPAM, hence allowing for the successful printing of the double-layer structures. The printed PEG layer was thoroughly washed with isopropyl alcohol (IPA) and dried in air before the PNIPAM layer was printed on the PEG layer. This was a crucial step to ensure that the PNIPAM layer attached to the PEG structure, which allowed for better control over the interfacial composition.

Two designs were chosen to study the self-folding of the double-layer polymer-based structures. The basic layout was a flat rectangular sheet consisting of a PEG layer and a PNIPAM layer of equal thickness (D1) (Figure 2B). For comparison, a sheet was also printed only with PEG (D2) and only with PNIPAM (D3). The thermal responsiveness of the sheets was tested by placing the swollen sheets in water at different temperatures (T ~ 50 and 15 °C). As expected, at 15 °C, all three sheets were flat. When placed in the hot water (T ~ 50 °C), the three sheets showed different responses. Sheet D2 did not show any shape/volume change, while sheet D3 rolled up along the longer side of the structure and shrank to a smaller size. Sheet D1, which was expected to have a combination of the responses shown by D2 and D3, remained unchanged in the hot water (Figure 2C). One possible reason for this could be that the PEG layer was too rigid to bend as a response to the volume change in the PNIPAM layer. The edges of the structures were not very smooth due to the very thin polymer layer, and making the structures slightly thicker resulted in smoother surfaces.

The design of the double-layer sheet needed slight modifications to obtain any geometric changes with temperature. Kirigami is a Japanese paper-cutting art that involves the addition of cuts in a paper sheet that allows for the pattern to stretch out upon applying a force at the ends of the paper. This kirigami-engineered elasticity is a technique that imparts a high degree of elasticity without stretching the material, but arises instead from the flexible geometries supported by the cuts. Many scientists are adapting this concept for developing highly stretchable structures for diverse applications, including stretchable electronic devices [50,51,52,53], soft robotic grippers [54] and actuators [55,56]. Zadpoor and co-workers recently reported the design of self-folding origami structures via a kirigami approach, allowing a double-layer sheet to fold upon the activation of a mechanical trigger [35].

To obtain thermally triggered self-folding systems, we adopted a similar kirigami-inspired double-layer concept. Structure D1 was altered by adding slits/cuts (parallel to the length of the sheet) only to the rigid backbone of the sheet, with no cuts in the PNIPAM layer (structure D4). The spacing between cuts was studied by Lamoureux et al., and was found to play a significant role in determining the stretchability of a sheet [57]. For optimum flexibility, we used a cut pattern similar to the one described by Zadpoor and co-workers, as shown in Figure 2B [35]. To observe how the folding behavior of the structure depends on the orientation of the cuts, another modification to the design was made. Structure D5 was constructed with the cuts aligned perpendicular to the length of the sheet (Figure 2B). After equilibrating in water at 15 °C, sheets D4 and D5 were both flat and swollen. Placing the kirigami sheets in hot water (T ~50 °C) caused both D4 and D5 to fold, but the direction of folding was atypical. Breger et al. reported on similar self-folding PNIPAM-based double-layer microgrippers that would fold in either direction, depending on the temperature of the medium. For instance, at T < LCST, the microgrippers would fold such that the swollen PNIPAM layer would face outwards [19]. In contrast, our kirigami sheets showed folding in opposite directions, with the PNIPAM as the outer layer when the structure was heated to T > LCST. A possible explanation for this unpredicted behavior could be that due to the presence of the cuts, the PEG layer became very flexible and could bear the stress, hence allowing the structure to fold with the PEG inward. The orientation of the cuts was also found to govern the line of symmetry along which the folding took place; for instance, structures D4 and D5 underwent folding in a direction perpendicular to the length of the cuts. Despite the sheets’ peculiar behavior, the cuts were successful in introducing flexibility into the structures and dictating the folding direction. The exact dimensions for the kirigami sheets are provided in Figure S1.

To study the degree of folding as a factor of the responsive–non-responsive layer thickness ratio, D4 was printed with varying PNIPAM and PEG layer thicknesses. Sheets D4a-c had PNIPAM:PEG thickness ratios of 1:3, 1:1 and 3:1, respectively. At a temperature of approximately 50 °C, the degree of folding of the sheets, measured using ImageJ.JS software, was found to increase with the increasing PNIPAM content of the structure, as anticipated (Figure 2C). The folding mechanism has been discussed in detail elsewhere in the literature [58,59,60], and we believe that the same mechanism applied to the models presented in this paper. Although good folding was obtained for the 3:1 PNIPAM:PEG thickness ratio, the overall structure was fragile, being mostly composed of PNIPAM. To improve the folding without compromising the mechanical properties of the structure, we proposed altering the thickness of the cuts based on an earlier report by Dickey and co-workers, who described the effect of changing the hinge width on the degree of folding [61]. Hence, we printed a modified bilayer sheet with a 1:1 PNIPAM:PEG layer thickness but with bigger cuts, while maintaining the gaps at the same ratio as the original models (D4 and D5) (exact dimensions are given in Figure S2), and a significant improvement was observed, with a folding of 330° obtained for the modified sheets (see Figure 2D and Supporting Videos S1 and S2). In our models, the hinges were made of PNIPAM, and the shrinkage of the PNIPAM at T > LCST caused the selective folding in the hinge area. Increasing the hinge area therefore increased the shrinkage, while the rest of the structure remained unchanged. This was observed as a greater folding with broader hinges for the overall object. An interesting observation was made when evaluating the thermal responsiveness of the D4 and D5 structures with larger cuts. Videos S1 and S2 show that the swollen structures instantly folded when dropped into hot water, in a direction parallel to the length of the cuts, with the PNIPAM layer outwardly oriented. Intriguingly, as the structure equilibrated, it opened up and started folding in the opposite direction, with the structure folded in a direction perpendicular to the length of the cuts and the PNIPAM layer facing inward. This observation can be explained by considering the thickness of the overall structure. When the sheet was dropped into the hot water, the outermost PNIPAM layers that were in direct contact with the heat reacted rapidly, leading to immediate shrinking and, hence, folding in the manner previously described. However, as the structure equilibrated, the inner PNIPAM portions started to shrink as the heat slowly diffused towards the core of the structure, causing the structure to become mechanically unstable and, thus, rearrange to the most stable geometry (see Supporting Video S3). The control of the extent of folding by varying the thickness of the cuts establishes one more function that can be used to fine-tune the folding behavior of such bilayer structures. Guo and co-workers conducted similar studies with models to predict the folding behavior of bilayer structures; however, the models were based on the use of different passive polymers with varying Young’s moduli to control the extent of the bending [60]. In this study, the same control can be obtained for a simple structure based on only two polymers by changing the thickness of the kirigami cuts.

**Figure 3:**
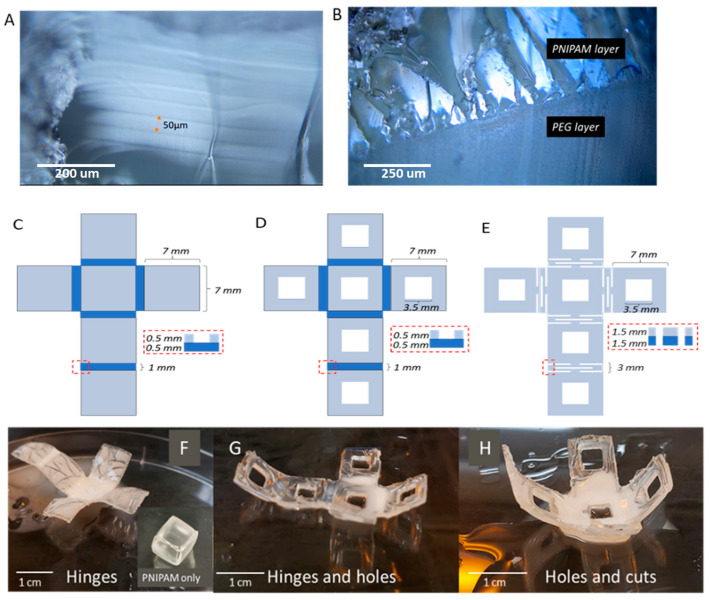
Optical microscopy images of PNIPAM cross section (**A**). PNIPAM/PEG cross-sectional interface (**B**). Sheet designs for self-folding boxes (**C**–**E**). 3D printed sheets in a collapsed state with various degrees of folding (**F**–**H**).

The microstructure analysis allowed for the analysis of the internal microstructure of the freeze-dried PNIPAM hydrogel (94 wt%). The internal architecture of PNIPAM/PEG mixed hydrogel systems was reported earlier by Lee et al., and they investigated the effect of changing the PNIPAM:PEG ratio and the molecular weight of PEG on the microstructure of the final hydrogel [62]. However, in this study we wanted to observe the microstructure of the interface to examine the strength of the adhesion of the PNIPAM to the PEG layer in our double-layer structure. The surface of the PNIPAM showed wrinkled patterns, typical of PNIPAM (Figure S2A) [63,64], while the cross-sectional image of the PNIPAM showed some pores (Figure S2B). The cross section of the PNIPAM was also captured by optical microscopy, as displayed in Figure 3A. The DLP printed layers can be observed, with each layer approximately 50 μm thick. The cross section of the double-layer structure was analyzed, and Figure 3B shows the difference between the two polymer layers and how well the layers adhered to each other, with no cracks between the two parts. The interfacial stability of the double-layer 3D printed objects was tested by reversibly heating and cooling the structures, and after more than 20 cycles, the layers did not peel apart. The double-layer structure was also left in water at room temperature for over three months and no change was observed, as both layers remained glued together. Mechanical testing was also performed (Figure S4); the interfacial tensile strength was stronger than the tensile strength in the PNIPAM, evident from the structural tearing in the PNIPAM portion instead of the at the interface between the PNIPAM/PEG.

Three-dimensional--printed self-folding boxes have been fabricated using different polymers and different stimuli to trigger a shape change [3,59,65]. In most cases, the printing was performed using a LaserJet printer or a direct ink writing method. To the best of our knowledge, 3D-printed self-folding boxes using a DLP printer have not been reported and hence, in this study, we wanted to use the DLP printing technique to generate self-folding boxes based on our new PNIPAM pre-polymer matrix. Our design was very similar to the reported ones and consisted of six panels linked via hinges to give a flat cross-shaped structure (Figure 3C). In the case of one polymer system, the cross was printed using only the 94 wt% PNIPAM. Upon increasing the temperature of the medium from room temperature to ~50 °C, the flat hydrated cross folds instantly into a box (Figure 3F inset).

Crosses of various dimensions were printed to obtain the optimum folding upon applying the thermal stimulus, and the best one is provided in Figure 3E. The PNIPAM crosses were very soft, and to make them stronger we applied the same principle as for the kirigami sheets and printed the cross as a double-layer system. Due to the cross design, we kept the same thickness for each layer. The sheets with hinges did not achieve sufficient folding in a hot state (Figure 3F). Attempts were made to modify the design of the sheet, first by cutting out holes to make the panels lighter and encourage folding (Figure 3D). Folding at higher temperatures was still not enough to form a closed box (Figure 3G). The second effort was made by replacing the hinges with kirigami cuts and increasing the “cut area” to increase flexibility and folding (Figure 3E). The high printing resolution of the DLP printer allowed for the addition of slits 0.5 mm wide, which resulted in the better folding of the sheet than the one with the hinges. Similar to the kirigami-inspired sheets, the thickness of the slits in the hinge area can be increased to improve the folding of the boxes, hence showing the potential of kirigami cuts in 4D printing (Figure 3H).

**Figure 4:**
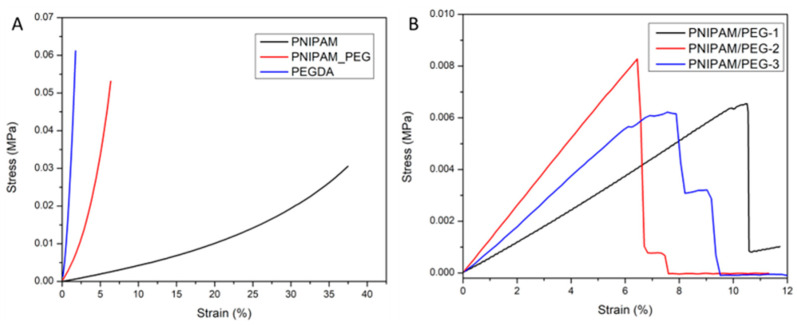
Stress vs. strain plots for PNIPAM, PEG and PNIPAM/PEG samples for compression (**A**) and tensile tests (**B**) measured on DMA850 TA instrument.

The mechanical properties of the single- and double-layer structures were evaluated using a dynamic mechanical analyzer, Discovery DMA850 (TA instrument 159 Lukens Drive, New Castle, DE 19720, USA) and an Instron Universal Testing System (Instron Corporation, 825 University Ave, Norwood, MA 02062-2643, USA) (Figures S3–S5). Compression and tensile tests were performed using the respective clamps and the single- and double-layer samples were prepared according to the specific dimensions. The mechanical properties were all measured in their swollen states (at room temperature), and the samples were allowed to equilibrate in water for 24 h before testing. The stress versus strain plots for the compression and tensile tests are shown in Figure 4, and the Young’s moduli derived from the plots are given in Table 1.

The static compression test was performed on the PNIPAM and PNIPAM-PEG systems and, as expected, the double-layer system had a higher Young’s modulus compared to that of the PNIPAM disc. Dynamic compression tests were also performed on the two single- and double-layer systems (Figures S6 and S7). The behavior of PNIPAM around its LCST is well established, and its improved mechanical properties with increasing temperature (T > LCST) are caused by the loss of water that makes the hydrogel stiffer than in its swollen state [66]. The slow response of PNIPAM to temperature is evident from the heating ramp rate. The slower ramp of 1 °C/min did not show any significant change in the modulus versus temperature plot. However, an increase in the storage modulus starting at around 35 °C was observed when the PNIPAM disk was heated at 3 °C/min (see Figure S6). A more significant gain in the storage modulus was expected at T > LCST, but due to the slow response of PNIPAM, the measurement was recorded on a hydrogel at a degree of deswelling lower than what was likely for a given temperature [66]. The same ramp was used for the double-layer disk, and the dynamic compression test also showed some increase in the storage modulus around 35 °C, but to a slightly lesser extent (0.023 MPa for the double layer versus 0.025 MPa for the PNIPAM single layer), as would be expected due to its lower PNIPAM content (Figure S7).

The tensile testing was performed in two different conditions to determine (1) the strength of adhesion between the PNIPAM and the PEG, using the DMA850 instrument, and (2) the strength of the overall double-layer system, measured on the Instron. The first sample was prepared for the static tensile testing and comprised a PNIPAM portion with PEG on either side of the PNIPAM (Figure S8B). The measurements were obtained at room temperature with the PNIPAM in the swollen state, and the plots are provided in Figure 4. The photograph of the sample after the tensile test shows that the cut in the PNIPAM, however, cracked, and most likely started at the interface between the two polymers. If the adhesion between the PNIPAM and the PEG was very weak, a clean cut at the interface would have been observed. However, since the cut only started at the interface and moved through the PNIPAM section, this suggests that the overall PNIPAM-PEG adhesion was stronger than that of the bulk PNIPAM. The second sample was prepared in a dog-bone shape according to ASTM D638 Type D. The double-layer sample was prepared with PNIPAM and PEG of equal thickness, while the single layer was prepared with only crosslinked PEGDMA. The plots obtained are presented in Figure S5B, and the difference in the PEG layer between the two samples is evident from the drop in Young’s modulus for the double-layer sample. The tensile data also confirm the strong PNIPAM-PEG adhesion, since both layers broke at the same time.

The experimental data were further supported by simulation studies using a COMSOL Heat Transfer modular guide, derived from the first law of thermodynamics. The parameters for the PNIPAM and PEG were chosen, and the design was built as a bilayer system with longitudinal and cross-sectional cuts in the PEG layer. The von Mises stress of the (Finite Element Method) FEM simulation of the bilayer systems resulted in a temperature range of 293.15–330.15 K (Figure S9). Folding over an axis perpendicular to the direction of the cuts was observed in both cases, which agrees with our experimental observations and with similar reports [62]. On the other hand, it is difficult for a COMSOL simulation to explain the reason why the folding was perpendicular to the cuts. More parameters, including the thermal expansion coefficient and the thermal conductivity of the polymers, are required to enable the software to provide more insight.

Our study has demonstrated a simple approach to fabricate self-folding structures by introducing double-layer polymer sheets with kirigami cuts. It is important to note that the mechanical properties of polymers play an important role in obtaining self-folding systems with desirable structural integrity. The observed folding with time indicates that a dynamic shape change can be achieved through the appropriate design and right choice of materials. Although studying the effects of the chemical composition on the self-folding performance is not the focus of this study, we believe that such an investigation will provide important information for guiding the design of self-folding structures, and may offer extra properties to these systems [67].

## 4. Conclusions

We have used high-resolution 3D printing technology to rapidly print multiple polymers monolithically. We have successfully customized the design by applying origami and kirigami art forms to obtain an optimum response upon the application of a stimulus. The PNIPAM/PEG double-layer sheets printed using a DLP printer showed good adhesion and improved mechanical behavior compared to pristine PNIPAM structures. The control over the direction and extent of the folding of the double-layer systems under varying temperatures was found to depend on the kirigami cuts, and could be tuned to obtain the desired responses. The structures were also tested for over 20 cooling–heating cycles and kept in water for several months, without any structural damages or even changes in the degree of folding, demonstrating their recyclability. The experimental results were supported by COMSOL modeling that successfully predicted the folding behavior of the double-layer systems. The thermo-responsiveness of the double-layer systems can be applied to more elaborate structures, and our attempt at producing a self-folding box with our PNIPAM/PEG system showed that kirigami cuts make the structures more flexible and more prone to fold into a box shape than the structures that have only origami hinges.

## Figures and Tables

**Table 1:** Young’s modulus data from compression and tensile tests using DMA.

Sample	Compression Young’s Modulus (kPa)	Tensile Young’s Modulus (kPa)
PEG	19.9	328 ± 15 ^1^
PNIPAM	0.4 ± 0.2	-
PNIPAM-PEG	1.2 ± 0.1	171 ± 69 ^1^

^1^ Data were measured using Instron.

## Data Availability

The original contributions presented in the study are included in the article/supplementary materials, further inquiries can be directed to the corresponding author.

## Supplementary Materials

The following supporting information can be downloaded at: https://www.mdpi.com/article/10.3390/ma17205028/s1. Figure S1. Kirigami sheet dimensions; Figure S2. SEM images of PNIPAM surface (A) and cross section (B); Figure S3. Compression testing on PNIPAM-only sample in static (A) and dynamic (B) modes; Figure S4. Static tensile strength testing on PNIPAM/PEGDA sample using DMA850; Figure S5. Static tensile strength testing on crosslinked PEGDMA dog-bone (ASTM D638-Type IV) using Instron (A) and stress versus strain plots of single- and double-layer systems (B); Figure S6. Dynamic compression plots of swollen PNIPAM with ramps of 1 and 3 °C/min; Figure S7. Dynamic compression plots of swollen PNIPAM/PEG with ramp of 3 °C/min; Figure S8. Compression testing sample dimensions (A) and tensile testing sample dimensions (B); Table S1. Printing parameters for PNIPAM and PEG layers on Asiga MAX X27 printer; Table S2. Cross-sectional cuts model is built in dimensions; Figure S9. Simulation results of heating impact on bilayer material (PEG-PNIPAM) with longitudinal and cross-sectional cuts in PEG layer. (a) Longitudinal cuts in top layer (PEG) at time 0 with temperature of 293.15 [K], (b) simulation results after 1 s of heating bilayer material to 330.15 [K], (c) cross-sectional cuts in top layer (PEG) at time 0 with temperature of 293.15 [K], and (d) simulation results after 1 s of heating bilayer material to 330.15 [K]; Table S3. Parameters used to define PNIPAM and PEG; Figure S10. Presentation of PNIPAM coefficient of thermal expansion function alpha_iso (T); Video S1. The folding along the cutting direction above LCST; Video S2. The structure returns to its original shape in water below LCST and folds when immersed in water above LCST; Video S3. The structure folds perpendicular to the cutting direction after it folds along the cutting direction. References [62,68,69,70,71,72,73] are cited in the supplementary materials.
