# Supplementary material for: Four-Dimensional Printing of Multi-Material Origami and Kirigami-Inspired Hydrogel Self-Folding Structures
Source: Materials (Basel). 2024 Oct 15;17(20):5028. doi: 10.3390/ma17205028 (PMC11509088; doi:10.3390/ma17205028)
Supplement: Supplementary file 1 [file materials-17-05028-s001.zip › materials-3244571-supplementary.pdf]

Supporting information

Content

Figure S1. Kirigami sheet dimensions -- 3

Figure S2. SEM images of PNIPAM surface (A), cross-section (B) ----------------------------3

Figure S3. Compression testing on PNIPAM only sample in static (A) and dynamic (B) modes --4

Figure S4. Static tensile strength testing on PNIPAM/PEGDA sample using DMA850 - 4

Figure S5. Static tensile strength testing on crosslinked PEGDMA dog-bone (ASTM D638-Type IV) using Instron (A), Stress versus strain plots of single and double layer systems (B) --- 5

Figure S6. The dynamic compression plots of swollen PNIPAM at ramp of 1 and 3 ^{o}C/min ---5

Figure S7. The dynamic compression plots of swollen PNIPAM/PEG at ramp of 3 ^{o}C/min --- 6

Figure S8. Compression testing samples dimensions (A), tensile testing sample dimensions (B)--6

Table S1. Printing parameters for PNIPAM and PEG layers on Asiga MAX X_{27} printer -7

Table S2. The cross-sectional cuts model is built in dimensions ------------------------------ 7

Figure S9. Simulation results of the heating impact on bilayer material (PEG-PNIPAM) with longitudinal and cross sectional cuts in the PEG layer, (a) Longitudinal cuts in the top layer (PEG) at time 0 with temperature 293.15[K] (b) Simulation results after 1 second of heating the bilayer material to 330.15[K], (c) Cross sectional cuts in the top layer (PEG) at time 0 with temperature 293.15[K] (d) Simulation results after 1 second of heating the bilayer material to 330.15[K] ---9

Table S3. Parameters used to define PNIPAM and PEG ---------------------------------10

Figure S10. Presentation of PNIPAM coefficient of thermal expansion function alpha_iso(T) --10

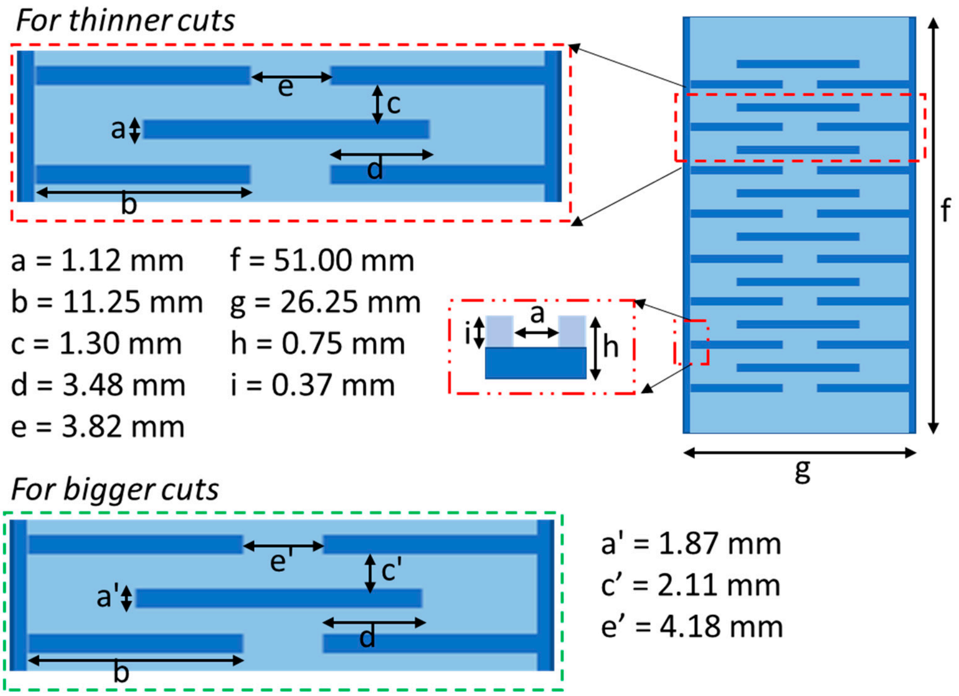

Figure S1. Kirigami sheet dimensions. The inset shows the z-dimension of PEC (bottom layer) and PNIPAM (top layer).

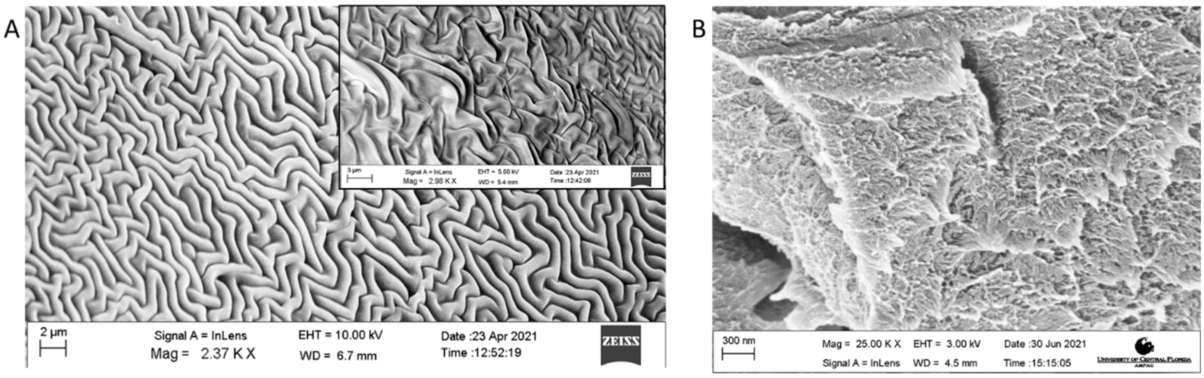

Figure S2. SEM images of PNIPAM surface (A), cross-section (B)

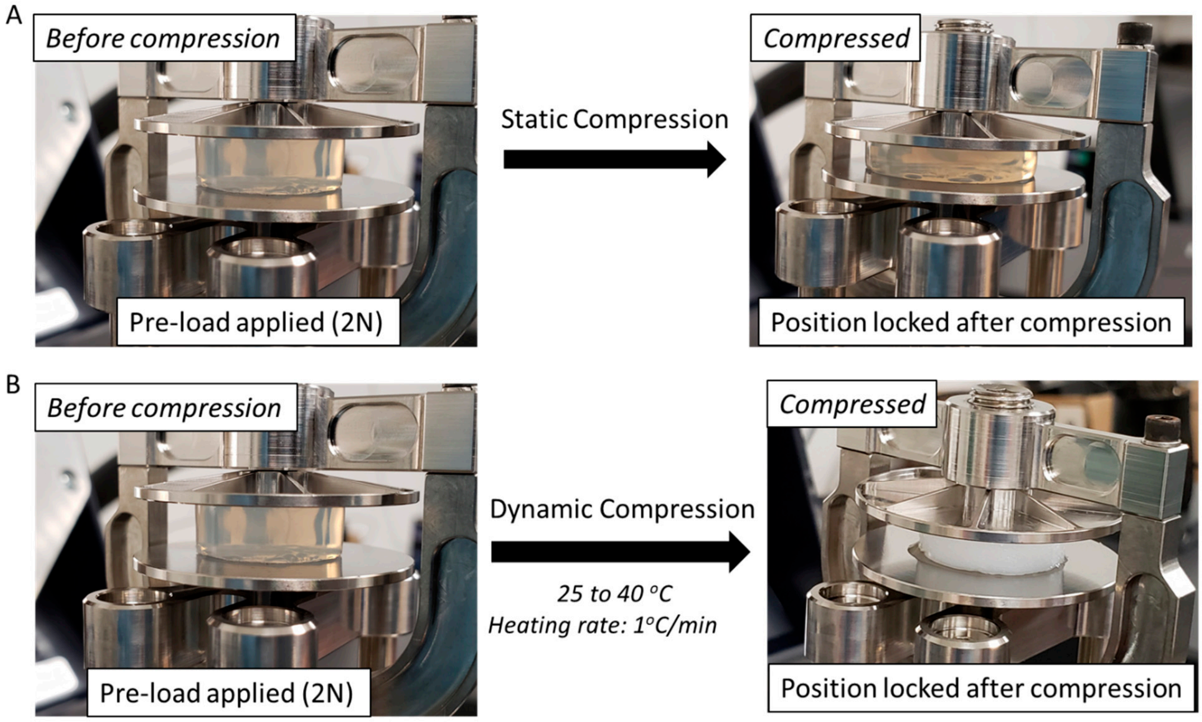

Figure S3. Compression testing on PNIPAM only sample in static (A) and dynamic (B) modes

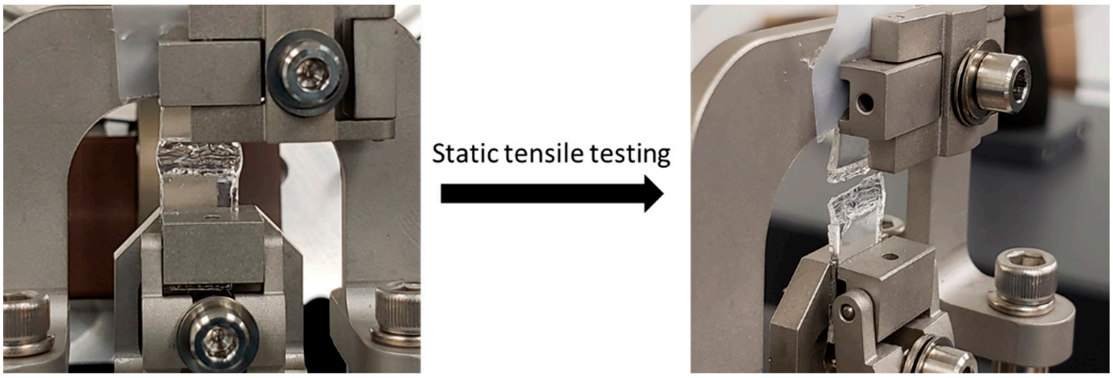

Figure S4. Static tensile strength testing on PNIPAM/PEG sample using DMA850

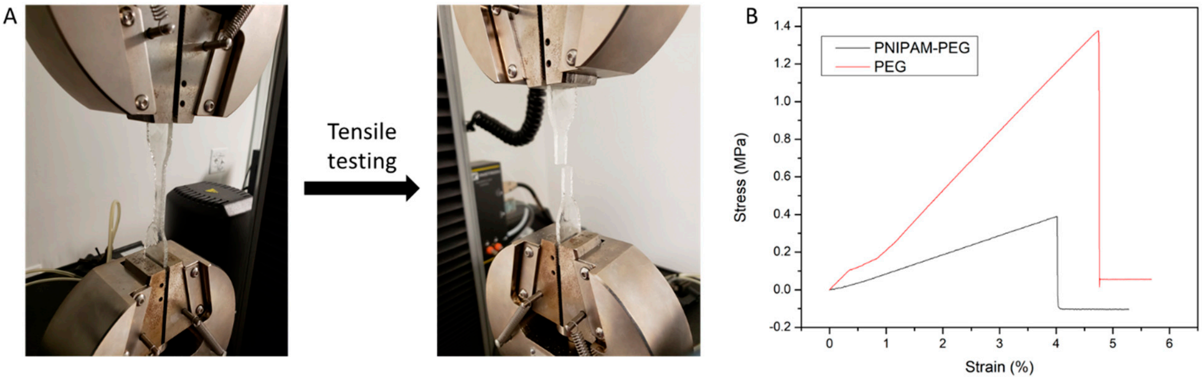

Figure S5. Static tensile strength testing on crosslinked PEGDMA dog-bone (ASTM D638-Type IV) using Instron (A), Stress versus strain plots of single and double layer systems (B)

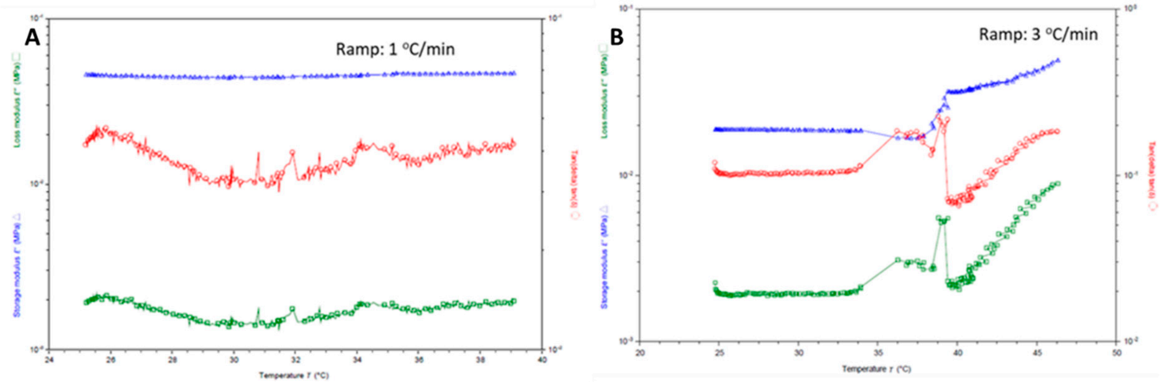

Figure S6. The dynamic compression plots of swollen PNIPAM at ramp of 1 °C/min (A) and 3 °C/min (B).

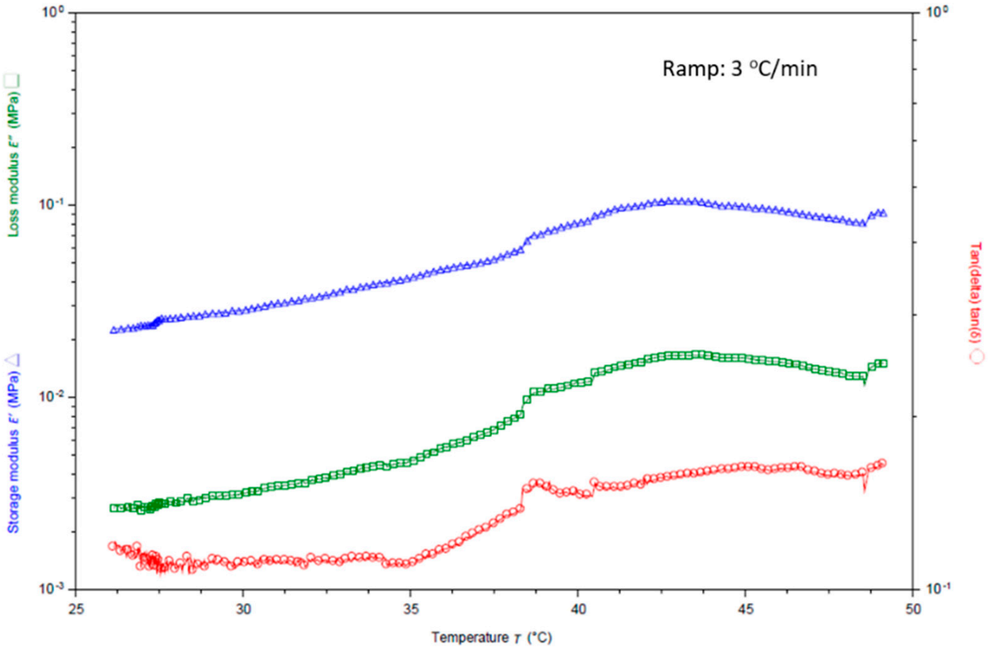

Figure S7. The dynamic compression plots of swollen PNIPAM/PEG at ramp of 3 °C/min

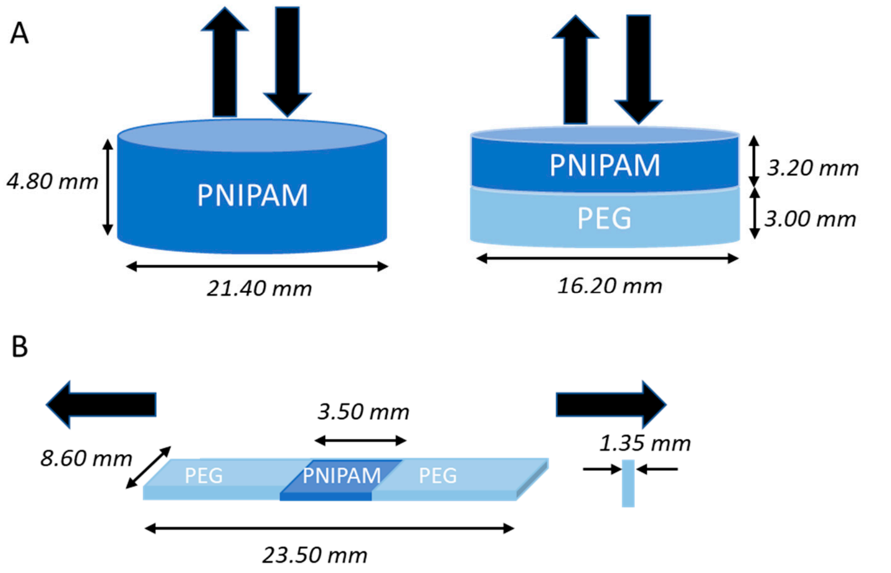

Figure S8. Compression testing samples dimensions (A), tensile testing sample dimensions (B)

Table S1. Printing parameters for PNIPAM and PEG layers on Asiga MAX X_{27} printer

Parameters	PEG layer	PNIPAM layer
Slice thickness (mm)	0.05	0.05
Exposure time (s)	1.5	10
Burn-in time (s)	1.5	10
Burn-in layers	1	1
Separation velocity	2	2
Separation distance	1.1	1.1
Approach velocity	2	2
Separation defect window	10	10
Separation defect window time	0.25	0.25

Table S2. The cross-sectional cuts model is built in dimensions

Sample Parameters	Expression	Value
PNIPAM thickness	0.5[mm]	5E-4 m
PEG thickness	0.5[mm]	5E-4 m
Length of sample, L1	45[mm]	0.045 m
Width of sample, W1	23[mm]	0.023 m
Length of the cut, L2	10[mm]	0.01 m
Width of the cut, W2	1.5[mm]	0.0015 m
Displacement a, da	1[mm]	0.001 m
Displacement b, db	2.5*da	0.0025 m

Derived from the first law of thermodynamics, the built-in equations, which are explained in the COMSOL Heat Transfer modular guide. Consider the conservation of energy where the changes in the Kinetic energy K and the Internal energy U over time t is caused by either the mechanical forces, P_{ext}, applied to the domain or the exchanged heat rate Q_{exch}.

$$\frac{\partial K}{\partial t} + \frac{\partial U}{\partial t} = P_{ext} + Q_{exch}$$

Considering $P_{ext} = \partial K/\partial t + P_{str}$, where P_{str} is the stress power which is converted into heat by dissipation, we can obtain:

$$\frac{\partial U}{\partial t} = P_{str} + Q_{exch}$$

P_{str} accounts for the Cauchy stress tensor and the strain rate tensor, whereas the heat rate exchange Q_{exch} accounts for the heat flux by conduction, radiation, and additional heat sources in the domain.

Localizing the heat balance equation after neglecting the heat flux by radiation, we get:

$$\varrho\, C_p \frac{\partial T}{\partial t} + \varrho\, C_p\, u \cdot \nabla T + \nabla .\, q = Q + Q_{ted}$$

Where, ϱ is the density (kg/m^3), Cp is the specific heat capacity at constant stress (J/(kg·K)), T is the absolute temperature (K), u is the velocity vector of translational motion (m/s), q is the heat flux by conduction (W/m^2), and Q represents additional heat sources (W/m^3). The equation solves for the thermoelastic damping, Q_{ted}, where later is used to identify the second Piola-Kirchhoff stress tensor and determine the deformation in material.

$$Q_{ted} = \alpha\, T\, \frac{\partial S}{\partial t}$$

Where α is the coefficient of thermal expansion (1/K) and S is the second Piola-Kirchhoff stress tensor (Pa).

In addition, the conductive heat flux variable, q, is evaluated using the temperature gradient ∇T and the effective thermal conductivity K_{eff} as follows:

$$q = -\, K_{eff}\, \nabla T$$

A comparison in the simulation results shown (Figure S9) represents the von Mises stress of FEM simulation to bilayer material on a temperature range 293.15(K) to 330.15(K). The simulation results over time explains the instantaneous folding due to the induced heat in the system. The folding direction is based on the cut's direction of the PEG layer. The bilayer system allows several opportunities to design target functions of a flexible device. Based on a simulation of the bilayer material, the coefficient of thermal expansion of PNIPAM is changing over temperature range.[1] The negative values of coefficient of thermal expansion causes a shrinkage of the PNIPAM layer in comparison to the PEG layer. In contrast to the cuts/extrusion in the hydrogel (PNIPAM) layer reported by Ma *et al.*,[1] the PNIPAM/PEG system has cuts in the PEG layer. Ma *et al.*, reported folding over an axis perpendicular to the cuts/extrusion direction in the PNIPAM layer. [1] Moreover, based on simulation results for cuts in the PEG layer, the simulation

results agree with the findings of Ma *et al.* of folding over an axis perpendicular to the direction of the cuts as shown in Figure S9.

The parameters used for the PNIPAM and PEG layers are provided in Table S3. Based on experimental results, relative assumptions were made in the COMSOL model to match the practical results. It is important to mention that changes in PNIPAM density with temperature is not considered to simplify the modeling. However, this results in smaller deflection in the simulation model in comparison to the practical experiment.

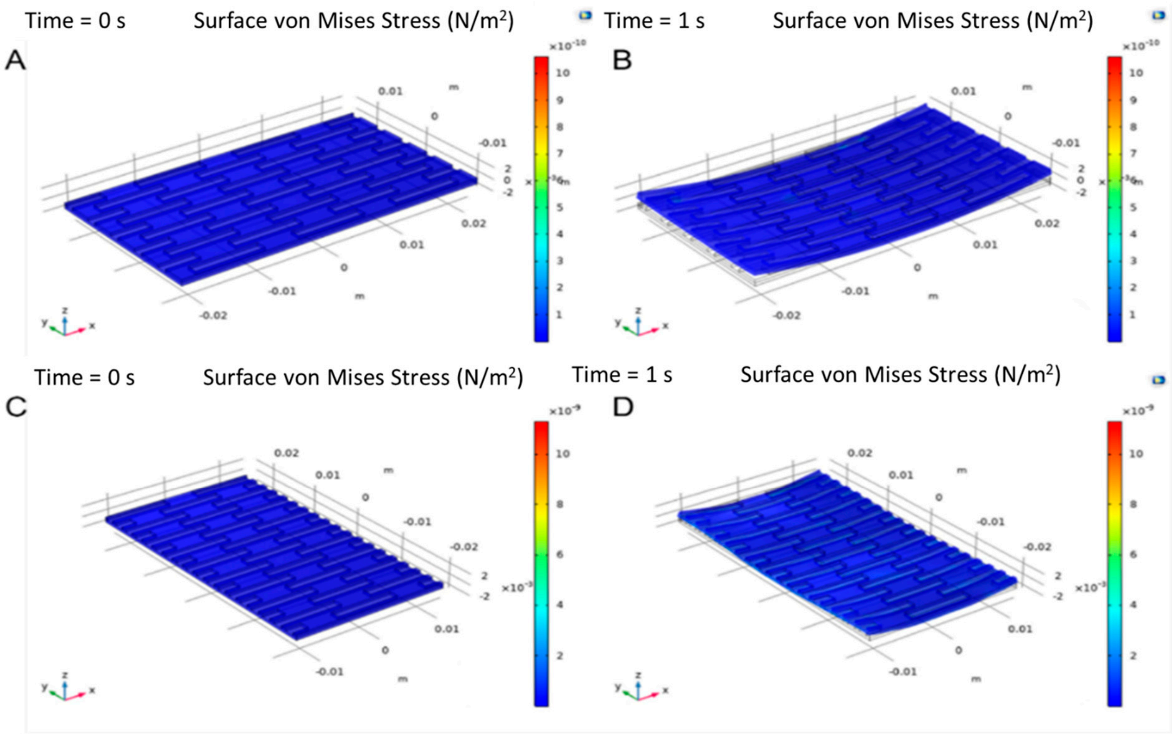

Figure S9. Simulation results of the heating impact on bilayer material (PEG-PNIPAM) with longitudinal and cross sectional cuts in the PEG layer, (a) Longitudinal cuts in the top layer (PEG) at time 0 with temperature 293.15[K] (b) Simulation results after 1 second of heating the bilayer material to 330.15[K], (c) Cross sectional cuts in the top layer (PEG) at time 0 with temperature 293.15[K] (d) Simulation results after 1 second of heating the bilayer material to 330.15[K]

Table S3. Parameters used to define PNIPAM and PEG

**Parameters**	**PNIPAM**	**PEG**
Density, ϱ	1386 [kg/m^3][1]	1209 [kg/m^3][2]
Heat capacity at constant pressure, C_p	40 [J/(kg·K)][1]	1558 [J/(kg·K)][3]
Thermal conductivity, *k*	0.35 [W/(m·K)][1]	0.316 [W/(m·K)][4, 5]
Coefficient of thermal expansion, alpha_iso	alpha_iso(T) [1/K][1]	4.2e-4 [1/K][2]
Young's modulus, E	1e4 [Pa]*	2e2[Pa]*
Poisson's ratio, nu	0.4[6, 7]	0.38*

* Assumed value based on model fitting results

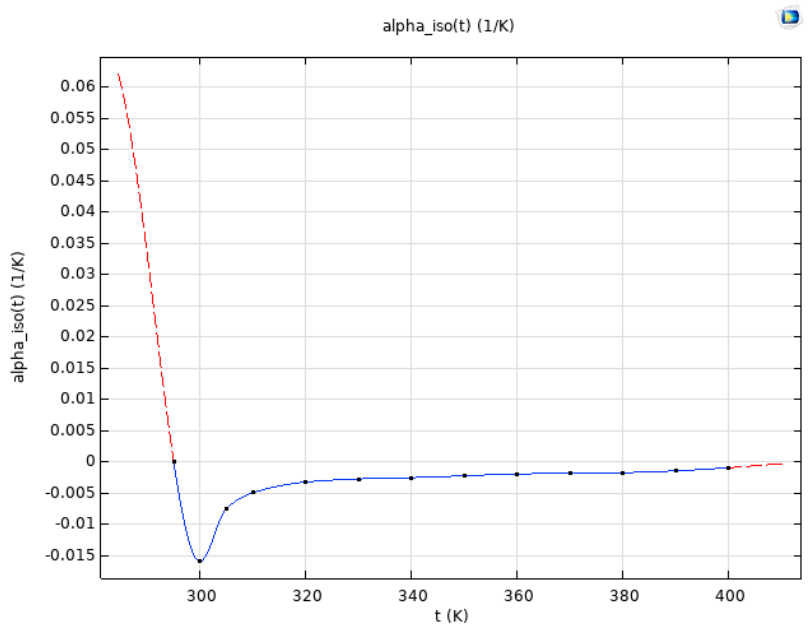

Figure S10. Presentation of PNIPAM coefficient of thermal expansion function alpha_iso(T). The red color and blue color reflect the positive and negative values of PNIPAM coefficient of thermal expansion function alpha_iso(T), respectively.

References

1. H. Ma and J. Zhou, 2021.
2. D. Sponseller and E. Blaisten-Barojas, *J. Phys. Chem. B*, 2021, **125**, 12892-12901.
3. Y. Kou, S. Wang, J. Luo, K. Sun, J. Zhang, Z. Tan and Q. Shi, *J. Chem. Thermodyn.*, 2019, **128**, 259-274.
4. L. He, H. Wang, H. Zhu, Y. Gu, X. Li and X. Mao, *Appl. Sci.*, 2018, **8**.
5. J. Yang, L.-S. Tang, R.-Y. Bao, L. Bai, Z.-Y. Liu, W. Yang, B.-H. Xie and M.-B. Yang, *Chem. Eng. J.*, 2017, **315**, 481-490.
6. N. Boon and P. Schurtenberger, *Phys. Chem. Chem. Phys.*, 2017, **19**, 23740-23746.
7. P. Voudouris, D. Florea, P. van der Schoot and H. M. Wyss, *Soft Matter*, 2013, **9**, 7158-7166.
